# Eighteen-year survival after GD2-directed Chimeric Antigen Receptor-Modified Immune Effector Cell Treatment for Neuroblastoma

**DOI:** 10.21203/rs.3.rs-4232549/v1

**Published:** 2024-04-11

**Authors:** Li Che-Hsing, Sandhya Sharma, Andras A. Heczey, David H.M. Steffin, Chrystal U. Louis, Bambi J. Grilley, Sachin G. Thakkar, Mengfen Wu, Tao Wang, Cliona M. Rooney, Malcolm K. Brenner, Helen E. Heslop

**Affiliations:** 1 Center for Cell and Gene Therapy, Baylor College of Medicine, Texas Children’s Hospital and Houston Methodist Hospital, Houston TX; 2 Department of Pediatrics, Center for Advanced Innate Cell Therapy, Baylor College of Medicine, Houston, TX; 3 Program in Immunology & Microbiology, Baylor College of Medicine, Houston, TX; 4 Dan L. Duncan Comprehensive Cancer Center, Baylor College of Medicine, Houston, TX

## Abstract

We report long-term outcomes up to 18 years of a clinical trial treating children with neuroblastoma with EBV-specific T lymphocytes and CD3-activated T cells – each expressing a first-generation chimeric antigen receptor targeting GD2 with barcoded transgenes to allow tracking of each population. Of 11 patients with active disease at infusion, three patients achieved a complete response that was sustained in 2, one for 8 years until lost to follow up and one for 18+ years. Of eight patients with a history of relapse or at high risk of recurrence, five are disease-free at their last follow-up between 10–14 years post-infusion. Intermittent low levels of transgene were detected during the follow up period with significantly greater persistence in those who were long-term survivors. In conclusion, patients with relapsed/refractory neuroblastoma achieved long-term disease control after receiving GD2 CAR-T cell therapy including one patient now in remission of relapsed disease for >18 years.

Genetic modification of immune effector cells with chimeric antigen receptors (CARs) to treat CD19 and BCMA positive malignancies represents a genuine advance in therapy with 6 products now approved by the FDA since 2017.^1,2^ However, follow up is relatively short with commercial products and recent reports of secondary T cell malignancies from the FDA^3^ suggest that longer-term safety concerns remain.^4^ Some investigational trials using CAR-T cell therapy to treat B-cell malignancy have reported up to a decade of disease remission^5,6^, but there are no published longer-term outcome reports of CAR-T cell therapy in solid tumors.

From 2004 to 2009 we conducted a phase 1 trial in children with neuroblastoma in which we infused two immune effector cell products – activated T cells expanded with OKT3 antibody (ATCs) and Epstein Barr Virus-specific T cells (VSTs) generated by stimulation with autologous EBV-transformed lymphoblastoid cell lines (LCLs). These were each transduced with first generation CARs recognizing GD2 expressed on neuroblastoma (NCT00085930).^7^ Each patient was treated with both CAR-ATCs and CAR-VSTs, that could be distinguished by real-time quantitative polymerase chain reaction (RT-qPCR) for separate non-coding nucleotides serving as barcodes. Thus each patient acted as a “self-control” excluding confounding factors and thereby allowing comparison between the behavior of ATCs and VSTs (Extended Data Fig. 1) The primary endpoint was the safety of the treatment with secondary endpoints of antitumor activity and the persistence of the two cell products. We reported the initial responses and subsequent follow up showing that the transgene in the periphery could be detected up to 192 weeks.^7,8^ Here we report the overall long-term clinical and biological outcomes with patients still being followed achieving 13 to 18 years of follow up.

Nineteen patients were enrolled in the study, of whom 11 (57.9%) had active relapsed disease (Table 1). Of the 8 patients (42.1%) with no evidence of active disease (NED), 5 had a history of relapsed disease and 3 were infused after completing therapy for high-risk disease. Demographic details are in Table 1. Of the 11 patients with active disease at time of infusion, 3 had complete responses, and 1 had a partial response (Extended Data Fig. 2). One of the patients with a complete response subsequently relapsed but two had sustained responses, one for 8 years until lost to follow up and one for 18+ years. Of the 8 patients with no evidence of active disease (NED) at the time of infusion, 5 were disease-free at their last follow-up between 10 – 14 years post-infusion. The event-free survival (EFS) at 15 years was 31.6% (patients with active disease: 18.2%; patients with NED: 50%; *p* = 0.044) (Fig. 1A and Extended Data Fig. 3A). The overall survival (OS) at 15 years was 36.8% (patients with active disease: 18.2%; patients with NED: 62.5%; *p* = 0.019) (Fig. 1B and Extended Data Fig. 3B).

Twelve of the 19 patients died between 2 months and 7 years post-infusion, all due to relapsed neuroblastoma. Seven patients are alive at the last follow-up with one loss of follow-up at 8 years, one withdrawing consent at 10 years, and the other 5 with continuing follow-up of 13+ to 18+ years. The patient who is alive at 18+ years post infusion had bone lesions prior to receiving CAR-T and attained a complete remission. She has never required any other therapy and is likely the longest-surviving patient receiving CAR-T therapy. Encouragingly, she has subsequently had 2 full-term pregnancies with normal infants. The overall toxicities observed during this long-term follow-up were attributable to previous chemotherapy, with sensorineural hearing loss as the most common adverse event (Table 1). One patient developed invasive ductal carcinoma of the breast 12 years after infusion, at age 32, attributed to her extensive chemotherapy and thoracic spinal radiation therapy prior to GD2 CAR infusion, both of which are well documented risk factors.^9^ We detected low levels of GD2 CAR transgene from ATCs (37.1 copy numbers/1 μg DNA) in the tumor sample likely representing long-persisting CAR-T cells, an observation consistent with reports that breast cancer stem cells may express GD2.^10^ The timeline for development of this secondary malignancy is consistent with her known prior chemotherapy and radiation exposure as well as our previous report noting that the 5-year cumulative incidence of a subsequent malignancy was not significantly different between recipients of genetically-modified immune effector cells and recipients of non-modified cells.^11^

After the first year of therapy, patients were followed annually, and peripheral blood was collected to detect transgenes derived from CAR-ATCs and CAR-VSTs. Eight of 19 patients (42.1%) still had detectable transgene of either one of the two CAR-T cell products beyond one year and the transgenic T-cells were still maintained in 5 of these 8 patients beyond 5 years (Fig. 1C). Interestingly, long-term survivors (LTS) had significantly longer persistence of detectable transgene compared to patients who were not LTS (*p* = 0.0002). (Fig. 1D). The low copy numbers in peripheral blood observed in the late time points may reflect the use of first-generation CAR constructs and the absence of lymphodepletion.

To date CAR-T cells have proven more effective in the treatment of hematological malignancies than of solid tumors, with a corresponding paucity of reports of even medium term clinical and biological outcomes in the latter. Trials of CAR-T cells in hematological malignancy report survival data ranging from 5–10 years.^5,6,12^ Here, we provide data on eight patients who survived more than 5 years after CAR-T cell treatment for a solid tumor, of whom 5 have follow-up from 14+ to 18+ years. Melenhorst et al. analyzed peripheral blood from some survivors at year 9 post-CD19-redirected CAR-T cells and found the long-existing CD4+ CAR-T cells still maintained their proliferative and cytotoxic abilities^6^ which correlates with our 5 year follow-up study in which persistence was highly concordant with the percentage of CD4+ cells and central memory T cells in the infused product.^8^ Although detection of transgene was intermittent and at a lower level in our study, likely reflecting a lack of a co-stimulatory domain in the construct, we still discovered a significantly longer transgene persistence in LTS, so that persisting CAR-T cells may contribute to long-term disease control.

Our study expressed a 1^st^ generation GD2 CAR in VSTs and ATCs. Nonetheless, the outcomes in patients with bulky relapsed/resistant disease and high relapse risk are comparable to other studies that treated neuroblastoma with GD2 CAR incorporating additional costimulatory signaling.^13–17^ The largest and most recent of these used autologous T-cells with both 4–1BB and CD28 costimulatory domains^15^ and our response rate, 3-year EFS, and OS are comparable with their results, and consistent with their observation that patients with low disease burden had significantly longer survival than those with a higher disease burden. Taken together, our data show the short- and medium-term benefits of CAR-T therapy in patients with neuroblastoma are also sustained long-term.

In conclusion, this study describes the longest reported survival after CAR-T therapy, demonstrating that GD2 CAR-ATC/VST therapy can safely produce up to 18+ years of complete remission in children with refractory/relapsed neuroblastoma, without evidence of malignancy or other long term sequalae attributed to CAR therapy.

## Methods

### Study design

**Extended Data Fig. 1.**
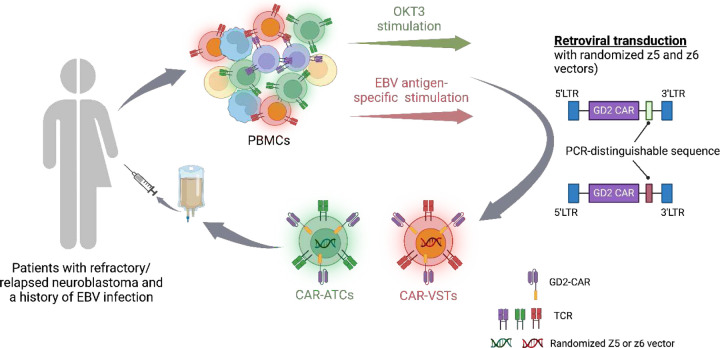
Graphical summary of the study design. NB, neuroblastoma.

The phase 1 clinical trial entitled, “Blood T Cells and EBV Specific CTLs Expressing GD-2 Specific Chimeric T Cell Receptors to Neuroblastoma Patients” (NESTLES; NCT00085930) was approved by the Baylor College of Medicine Institutional Review Board (H-13149), the Recombinant DNA Advisory Committee, the Institutional Biosafety Committee, and the FDA. The summary of the study design is demonstrated in Extended Data Fig. 1. Written informed consent was recollected when patients became older than 18 years old. The 5-year outcomes of all the 19 enrolled patients were described in our previous publications.^7,8^ This current report focuses on the very-long-term follow-up results including safety, efficacy, and T cell persistence.

### Clinical safety and outcome assessment

The event-free survival, or EFS, was defined from the date of the first GD2 CAR-T cell product infusion to the date of disease progression or relapse or last follow-up. OS was defined from the date of the first product infusion to the date of death from any cause or last follow-up. An LTS was defined as a patient who remained alive for over 5 years. All the complications and other medical issues during follow-up were collected using our standard operating process for long term follow up..

### Transgene copy number assessment.

RT-qPCR was conducted on patients’ peripheral blood mononuclear cells (PBMCs) isolated at various pre-defined time points to evaluate the presence and duration of CAR-ATCs and CAR-VSTs post-infusion. The two distinguishable retroviral constructs have a unique 12-base pair sequence, zeta 5 and zeta 6 respectively, as a barcode between the receptor stop codon and the 3’ long terminal repeat. The two immune effector cells were randomized to be transduced with either one of the two constructs, so they could be detected separately in patients’ PMBCs. Unless limited by sample availability, each sample was run in triplicate, alongside non-transduced cells (PBMCs or OKT3 activated PBMCs) that were used as negative controls. In this manuscript, the detection values are reported as copy numbers per 1 μg DNA. The detection levels were evaluated using a standard curve encompassing copy numbers of the gene of interest, ranging from 3 to 300,000 per 1 μg DNA.

In previous publications, the data calculated from the RT-qPCR was presented as the percentage of transgene-expressing cells determined based on a standard curve representing the percentage of transgene-constituting cells ranging from 0% to 100% transduced cells.^7,8^ The detection levels recorded as 100% of the standard correspond to 608 copies per ng, while the 1% standard corresponds to 6.08 copies per ng DNA respectively.

### Statistical analysis

The EFS and OS were calculated using the Kaplan-Meier method and compared by the log-rank test. Response rates were calculated using summary statistics.. Duration of detectable transgene was compared between LTS and non-LTS using the Mann-Whitney U test. A *p* value < 0.05 was considered as the statistically significant level.

## Extended Data

**Extended Data Fig. 2.**
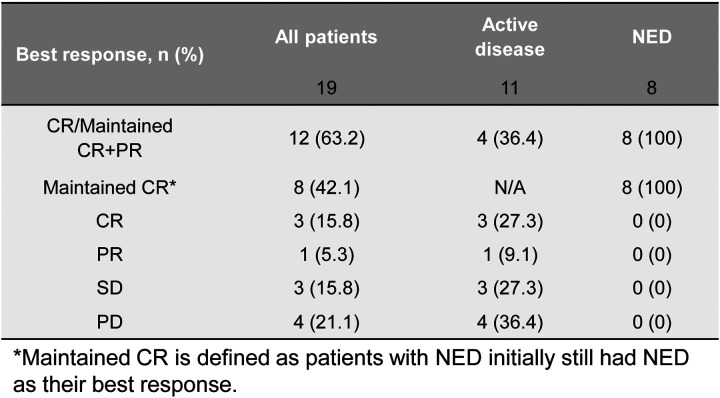
Table summary of patients’ best response during the therapy.

**Extended Data Fig. 3.**
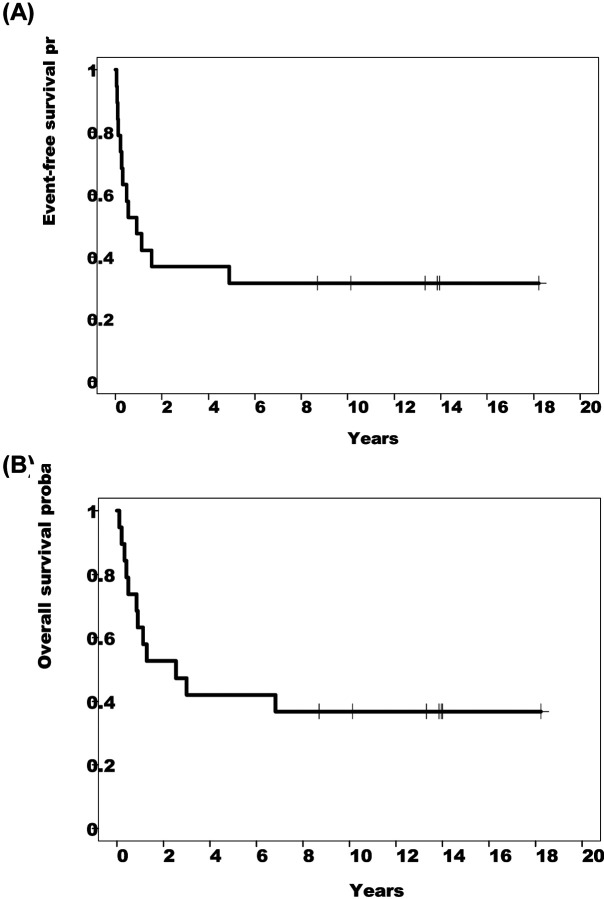
Survival estimates for all patients (n=19). (A) EFS. (B) OS.

## Figures and Tables

**Fig. 1.**
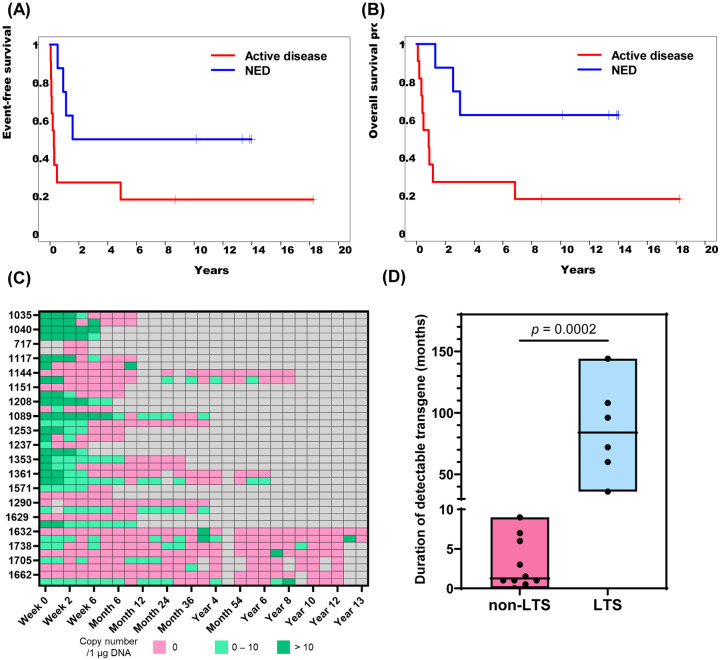
Long-term outcomes and T cell persistence after GD2 CAR-T infusions. (A) The EFS was defined from the date of first infusion to the date of disease relapsed or progressed, or last contact. (B) The OS was defined from the date of first infusion to the date of death with any causes or last contact. Log-rank test was used to compare the two groups. (C) The transgene levels (copy number/ 1 μg DNA) of both CAR-ATCs and CAR-VSTs in patients’ peripheral blood after infusion were measured by RT-qPCR and visualized using a heatmap. Each patient’s results (upper: CAR-VSTs; lower: CAR-ATCs) were presented in three categories: dark green, > 10 copy numbers; light green, 0 – 10 copy numbers; pink, undetectable. The gray color means there was no sample collected. (D) Difference in duration of detectable transgene between non-LTS and LTS. The median and range was demonstrated as the boxes. Three patients were excluded in the analysis (CAGT #1040 died shortly without providing accurate duration; #1089 and #1290 were lost to follow-up). Mann-Whitney U test was analyzed to compare the median in LTS and non-LTS.

**Table 1.** Patient demographics. The three dose levels were 2×10^7^ cells/m^2^; 5×10^7^ cells/m^2^; 1×10^8^ cells/m^2^. LTS, long-term survivors, defined as patients who remained alive for more than 5 years. NED, no evidence of active disease. FU, follow-up. PD, progression disease or disease relapse. SD, stable disease. PR, partial response. CR, complete response. NB, neuroblastoma.

Dose level	CAGT no.	Sex	Age at infusion	Stage	Disease burden at infusion	Best response	Last disease status	LTS	Status	Long-term complications
1	717	M	11	IV	Relapsed, bulky	PD	PD	No	Dead (0.8 y)	-
	1040	M	10	IV	Relapsed, bulky	PD	PD	No	Dead (0.1 y)	-
	1144	F	4	IV	Refractory, bone lesion	CR	CR	Yes	Alive (18.2 y)	Influenza B infection, adrenal insufficiency, ganglioneuroma
	1571	F	4	IV	Relapsed, bone lesion	PD	PD	No	Dead (0.3 y)	-
	1290	F	9	IV	Relapsed, bone lesion	CR	CR	Yes	Loss of FU (8.7 y)	-
	1632	F	20	IIa	NED	NED	CR	Yes	Alive (14.0 y)	NB relapsed multiple times but cancer free now, blood clot, grade 3 Her2(+) invasive ductal carcinoma of breast
	1738	M	5	IV	NED	NED	NED	Yes	Alive (14.0 y)	Bilateral sensorineural hearing loss
	1705	M	4	IV	NED	NED	NED	Yes	Alive (13.9 y)	Bilateral sensorineural hearing loss, short stature
	1662	M	9	IV	NED	NED	NED	Yes	Alive (13.3 y)	Mild scoliosis, bilateral sensorineural hearing loss
	1629	M	7	IV	NED	NED	PD	No	Dead (1.3 y)	-
2	1117	F	9	IV	Relapsed, bulky	PD	PD	No	Dead (0.9 y)	-
	1151	F	10	IV	NED	NED	PD	No	Dead (3.0 y)	-
	1035	F	15	IV	Relapsed, bone marrow	CR	PD	No	Dead (0.4 y)	-
	1208	M	3	IV	Relapsed, bulky	SD	PD	No	Dead (0.5 y)	-
	1253	F	9	III	Relapsed, bulky	Tumor necrosis, SD	PD	No	Dead (1.1 y)	-
	1089	F	4	IV	NED	NED	NED	Yes	Loss of FU (10.1 y)	Bilateral sensorineural hearing loss
3	1237	F	4	IV	Relapsed, bulky	Tumor necrosis, SD	PD	No	Dead (0.2 y)	-
	1353	M	7	IV	NED	NED	PD	No	Dead (2.5 y)	-
	1361	M	7	IV	Relapsed, bulky	PR	PD	Yes	Dead (6.8 y)	-

## Data Availability

The data that support the findings of this study are available from the corresponding author upon reasonable request.
